# Sex-specific implications of inflammation in covert cerebral small vessel disease

**DOI:** 10.1186/s12883-024-03730-z

**Published:** 2024-06-27

**Authors:** Bo-An Chen, Wei-Ju Lee, Lin-Chieh Meng, Yi-Chin Lin, Chih-Ping Chung, Fei-Yuan Hsiao, Liang-Kung Chen

**Affiliations:** 1 Department of Neurology, Taipei City Hospital Renai Branch, Taipei, Taiwan; 2 Program in Molecular Medicine, National Yang Ming Chiao Tung University, Taipei, Taiwan; 3 Center for Healthy Longevity and Aging Sciences, National Yang Ming Chiao Tung University, Taipei, Taiwan; 4 Department of Family Medicine, Taipei Veterans General Hospital Yuanshan Branch, Yi-Lan, Taiwan; 5 Graduate Institute of Clinical Pharmacy, College of Medicine, National Taiwan University, Taipei, Taiwan; 6 Department of Neurology, Neurological Institute, Taipei Veterans General Hospital, Taipei, Taiwan; 7 Department of Pharmacy, National Taiwan University Hospital, Taipei, Taiwan; 8 Center for Geriatrics and Gerontology, Taipei Veterans General Hospital, Taipei, Taiwan; 9 Taipei Municipal Gan-Dau Hospital (Managed by Taipei Veterans General Hospital), Taipei, Taiwan

**Keywords:** Cerebral small vessel disease, Sex disparity, Homocysteine, Inflammatory marker, vascular inflammation

## Abstract

**Background:**

The relationship between inflammation and covert cerebral small vessel disease (SVD) with regards to sex difference has received limited attention in research. We aim to unravel the intricate associations between inflammation and covert SVD, while also scrutinizing potential sex-based differences in these connections.

**Methods:**

Non-stroke/dementia-free study population was from the I-Lan longitudinal Aging Study. Severity and etiology of SVD were assessed by 3T-MRI in each participant. Systemic and vascular inflammatory-status was determined by the circulatory levels of high-sensitivity C-reactive protein (hsCRP) and homocysteine, respectively. Sex-specific multivariate logistic regression to calculate odds ratios (ORs) and interaction models to scrutinize women-to-men ratios of ORs (RORs) were used to evaluate the potential impact of sex on the associations between inflammatory factors and SVD.

**Results:**

Overall, 708 participants (62.19 ± 8.51 years; 392 women) were included. Only women had significant associations between homocysteine levels and covert SVD, particularly in arteriosclerosis/lipohyalinosis SVD (ORs[95%CI]: 1.14[1.03–1.27] and 1.15[1.05–1.27] for more severe and arteriosclerosis/lipohyalinosis SVD, respectively). Furthermore, higher circulatory levels of homocysteine were associated with a greater risk of covert SVD in women compared to men, as evidenced by the RORs [95%CI]: 1.14[1.01–1.29] and 1.14[1.02–1.28] for more severe and arteriosclerosis/lipohyalinosis SVD, respectively. No significant associations were found between circulatory hsCRP levels and SVD in either sex.

**Conclusion:**

Circulatory homocysteine is associated with covert SVD of arteriosclerosis/lipohyalinosis solely in women. The intricacies underlying the sex-specific effects of homocysteine on SVD at the preclinical stage warrant further investigations, potentially leading to personalized/tailored managements.

**Trial registration:**

Not applicable.

**Supplementary Information:**

The online version contains supplementary material available at 10.1186/s12883-024-03730-z.

## Background

Cerebral small vessel disease (SVD) represents a discernible outcome of neurovascular aging, characterized by two distinct pathologies: arteriosclerosis/lipohyalinosis and cerebral amyloid angiopathy (CAA) and several neuroimaging features: white matter hyperintensity (WMH), lacunes, cerebral microbleeds (CMB), dilated perivascular space (PVS) and cortical superficial siderosis [1–7]. SVD is not only an age-related neuroimaging biomarker but also a significant etiological contributor to major neurological diseases that afflict the older population, such as 25% of ischemic stroke, 80% of hemorrhagic stroke, and 45% of dementia.^1^ Though its clinical significance, the biological mechanisms behind SVD, particularly at its early, preclinical stage, is largely unknown [2]. 

Increasing evidence from clinical studies has suggested inflammation as a factor involved in the pathophysiology of SVD [3–5]. Studies have identified significant correlations between circulatory inflammation markers and SVD, however more evident in stroke cohorts as opposed to the healthy, stroke- and dementia-free population. It is worth noting, however, that the majority of these investigations examined only a single SVD marker, with WMH being the most frequently studied, and the precise role of inflammation in various etiologies of SVD remains a subject of ongoing inquiry [3, 8]. Differences in immunological development and responses between men and women have given rise to varying susceptibility to detrimental inflammatory reactions [6, 7]. In addition, sex difference has been observed in the link between numerous vascular risk factors and cerebrovascular disease [9]. However, the extent to which sex modulates the relationship between inflammation and SVD remains unanswered. In light of the aforementioned lack of understanding, the current investigation endeavors to explore possible sex-based dissimilarities in the correlation between inflammation and SVD, encompassing the severity and underlying causes of SVD, in a community-dwelling population that is free from dementia and stroke. We hypothesize that the association between inflammation and SVD may vary between women and men. The findings of this study may advance our comprehension of the mechanisms involved and aid in developing personalized therapeutic interventions in the early, preclinical stages of SVD.

## Methods

### Study population

**Fig. 1:**
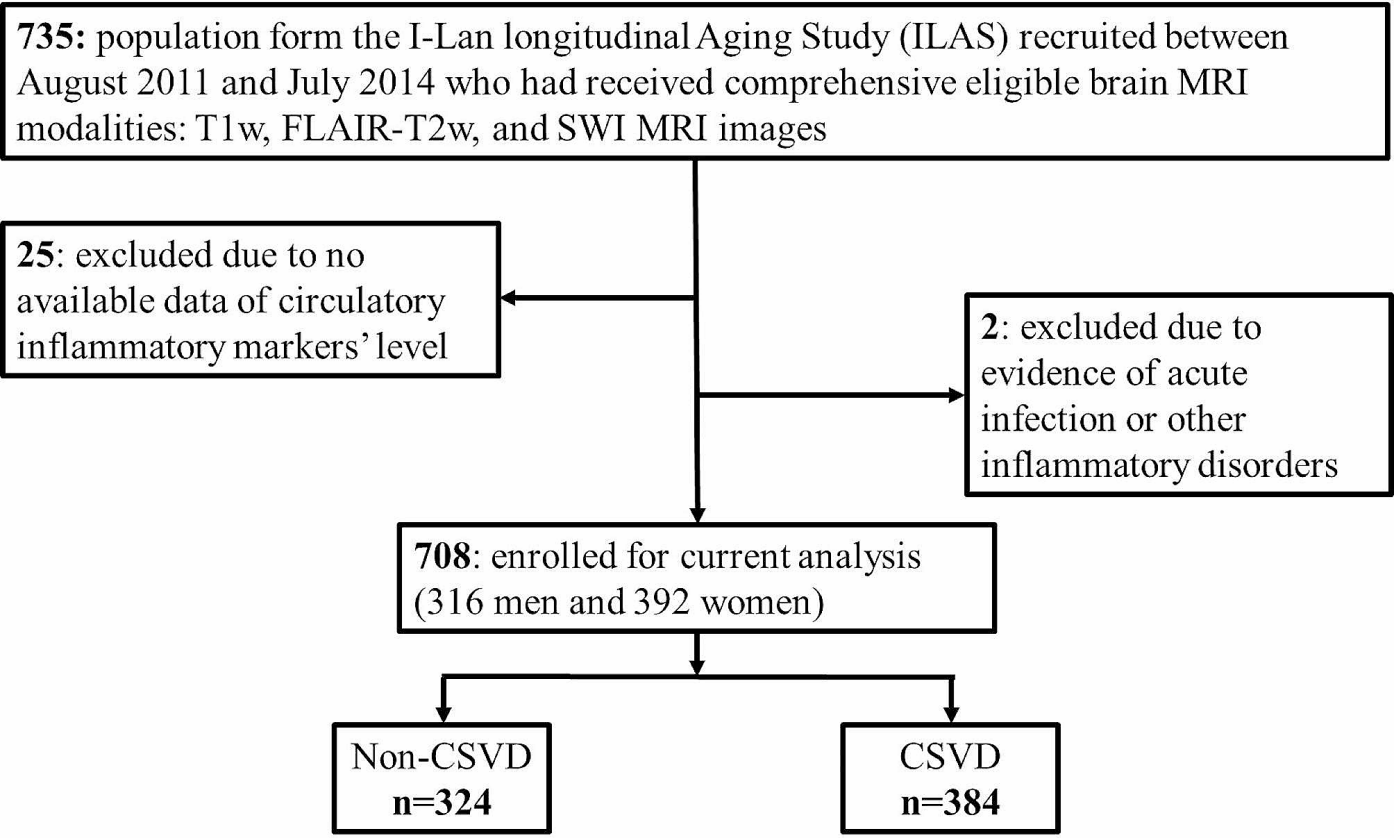
The overview of data recruitment

Figure 1 provides an overview of the data recruitment process. Participants were from the I-Lan Longitudinal Aging Study (ILAS), an ongoing community-based longitudinal cohort study that recruited individuals aged ≥ 50 years in the I-Lan County of Taiwan. Detailed information about the recruitment criteria and collected data for ILAS can be found in prior research publications [10]. To assess the early, preclinical stage of SVD, participants who had undergone comprehensive brain MRI for SVD assessment were included, while those with neuropsychiatric disorders such as dementia, major depression, stroke, and brain tumors were excluded. Additionally, individuals with acute infections or other inflammatory disorders, indicated by a white blood count of over 10 × 10^3^/mm^3^ and a high-sensitivity C-reactive protein (hsCRP) level exceeding 0.5 mg/dL, were also excluded.

### Vascular risk factors

The record and definitions of vascular risk factors are provided in the Supplementary Material.

### Biochemistry assessment for the levels of circulatory inflammatory biomarkers

Upon a 10-hour overnight fasting, serum samples for homocysteine and hsCRP measurements were collected from study participants. Homocysteine levels were determined by utilizing an automated chemiluminescence immunoassay system (ADVIA®, Siemens, Germany), whereas hsCRP levels were assessed by employing an automatic chemistry analyzer system (ADVIA®, Siemens, Germany).

### Brain MRI acquisition

The acquisition of multimodal neuroimaging data was carried out at the National Yang Ming Chiao Tung University to obtain information about SVD markers, which included WMH, lacunes, and CMBs. The details are provided in the Supplementary Material.

### Volume quantification of WMH and assessment of other MRI SVD markers

We used a quantification method for WMH assessment and visual scales for the other SVD markers, such as lacune and CMB. Detailed methods are provided in the Supplementary Material.

### SVD types (Arteriosclerosis/lipohyalinosis or CAA)[11]

**Fig. 2:**
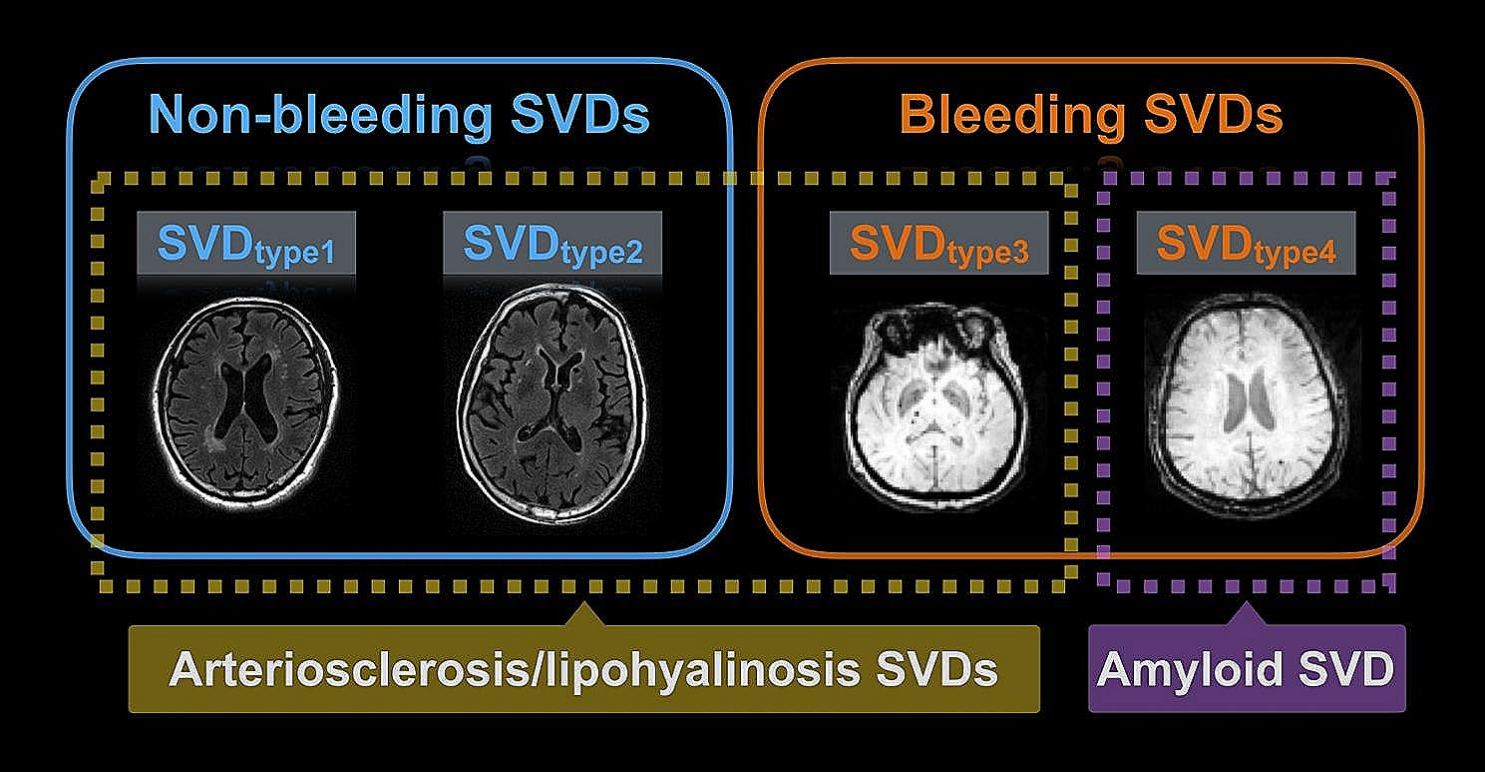
The classification of covert cerebral small vessel disease (SVD) SVD type 1 = isolated white matter hyperintensities (WMH; ≥ 50th WMH volume/total intracranial volume ratio in the total population = 0.7 × 10^− 3^); SVD type 2 = WMH with lacune(s) but without cerebral microbleed (CMB); SVD type 3 = mixed CMB; SVD type 4 = strictly-lobar CMB.

The categorization of SVD was carried out using a hierarchical approach comprising three consecutive steps. Firstly, the presence or absence of CMBs was determined. Secondly, the severity of WMH was assessed based on whether it exceeded the 50th percentile of the WMH/ total intracranial volume (TIV) ratio (used to define severe WMH in the present study). Finally, if CMBs were present, the combination of lacunes with severe WMH and the location of CMB (mixed or strictly lobar) were considered. The resulting classification divided SVD into four types: SVD types 1 and 2 represent non-bleeding SVD, where WMH was present with or without lacunes, respectively, while SVD types 3 and 4 were associated with bleeding. Specifically, mixed or strictly lobar CMB was designated as SVD types 3 and 4, respectively. This stratification scheme has been validated in our previous study, demonstrating its capability to reveal distinct clinical manifestations and neuroanatomic alterations in SVD type 4, including changes in gray matter volume and white matter microstructure [11]. SVD types 1, 2, and 3 exhibited similar patterns but with varying severity of neuroanatomic and clinical manifestations [11]. These findings suggest that the underlying etiology of SVD type 4 differs from the other three SVD types (type 1, 2, and 3). Based on our previous study, SVD type 4 is deemed attributing to CAA pathogenesis, while SVD types 1, 2, and 3 are categorized as arteriosclerosis/lipohyalinosis pathogenesis (as illustrated in Fig. 2) [11]. 

### SVD burden (Severity of SVD)

To gauge the extent of SVD severity in each participant, we computed SVD score that ranged from 0 to 3. Each point was allocated for the existence of severe WMH, CMB, or any lacune [12, 13]. 

### Statistical analysis

The analysis of group differences in continuous variables was conducted by means of non-parametric Mann-Whitney U test or Kruskal-Wallis test followed by post-hoc analysis. For categorical variables, we employed chi-square or Fisher’s exact tests. To explore the sex-specific relationship between SVD and each potential risk factor, we conducted sex-stratified multivariate logistic regression analysis and calculated the sex-specific odds ratio (OR). Thus, the outcomes were the presence of SVD of each severity and etiology, with inflammatory markers as predictors (i.e. homocysteine and hsCRP) and the other potential SVD risk factors (i.e. hypertension, diabetes mellitus, dyslipidemia, cigarette smoking habit) as confounders. An interaction term was added to the model to obtain the women-to-men ratio of ORs (RORs) and 95% CIs, which were used to evaluate whether the OR differed between the sexes [14]. Statistical analysis was conducted using the commercially available software SPSS 22.0. A p value less than 0.05, with two-tailed testing, was considered to indicate statistical significance.

## Results

The present study included 708 participants, consisting of 316 men and 392 women, as depicted in Fig. 1; Table 1. Dyslipidemia was found to be more prevalent in women compared to men, while smoking was reported more by men. Notably, the level of the circulatory vascular inflammatory marker, homocysteine, was significantly higher in men (median [interquartile range]: 13.60 [11.80–16.20] umol/L) than in women (10.75 [9.10-12.78] umol/L). The systemic inflammatory marker, hsCRP, was not found to be significantly different between the two sexes.

**Table 1:** Comparisons of demographics and profiles of SVD and inflammatory markers between sex

		Total *N* = 708		Male *n* = 316		Female *n* = 392		*P* value
Age, years		62.19 ± 8.51		62.82 ± 8.89		61.96 ± 8.16		0.151
HTN		245 (34.6%)		106 (33.5%)		139 (35.5%)		0.634
DM		100 (14.1%)		41 (13.0%)		59 (15.1%)		0.449
Dyslipidemia		74 (10.5%)		19 (6.0%)		55 (14.0%)		< 0.001
Cigarette smoking		106 (15.0%)		96 (30.4%)		10 (2.6%)		< 0.001
Simple SVD score								0.060
1		276 (39.0%)		135 (42.7%)		141 (36.0%)		
2 or 3		108 (15.3%)		52 (16.5%)		56 (14.3%)		
SVD type								0.206
1		246 (34.7%)		119 (37.7%)		127 (32.4%)		
2		47 (6.6%)		22 (7.0%)		25 (6.4%)		
3		56 (7.9%)		29 (9.2%)		27 (6.9%)		
4		35 (4.9%)		17 (5.4%)		18 (5.6%)		
Homocysteine, umol/L		12.10 (10.10–14.60)		13.60 (11.80–16.20)		10.75 (9.10-12.78)		< 0.001
hsCRP, mg/dL		0.064 (0.025–0.158)		0.056 (0.025–0.156)		0.066 (0.026–0.159)		0.516

Continuous variable: Mann–Whitney U test; Category variable: Fisher’s exact test or Pearson’s chi-squared test; N (%) or mean ± standard deviation or median (interquartile range)

DM: diabetes mellitus; HTN: hypertension; hsCRP: high-sensitivity C-reactive protein; SVD: cerebral small vessel disease

The severity of WMH in our criteria to define severe WMH (≥ 50th WMH volume/TIV in the total study population) was ≧ 0.7 × 10^− 3^, which was much milder than the criteria using visual Fazekas scale (Fazekas 2–3 = WMH/TIV ratio > 0.2–0.5%). There were 384 participants defining as the SVD population. Among them, 276 had mild SVD (SVD score = 1) and 108 had more severe SVD (SVD score = 2 or 3). For the stratification based on SVD pathogenesis, 246 people had type 1 SVD (non-bleeding SVD: isolated WMH), 47 people had type 2 SVD (non-bleeding SVD: WMH with lacune), 56 people had type 3 SVD (bleeding SVD: mixed CMB) and 35 people had type 4 SVD (bleeding SVD: strictly lobar CMB). Therefore, 349 (90.9%) of SVD population was indicated having arteriosclerosis/lipohyalinosis SVD while 35 (9.1%) was defining as CAA. There was no difference between men and women in terms of the severity or type of SVD (Table 1).

### Risk factors of coverts SVD in the total study population

We also conducted a comparison between demographic or clinical variables and SVD severities. Our findings revealed that age, as well as the proportions of hypertension, diabetes mellitus (DM), and dyslipidemia, were significantly different between SVD severity groups (Table 2, **left**). a. Specifically, individuals with more severe SVD were older and exhibited a higher likelihood of hypertension (no SVD vs. mild SVD vs. more severe SVD = 23.1% vs. 39.5% vs. 56.5%) and DM (7.1% vs. 18.8% vs. 23.1%), but were less likely to have dyslipidemia (13.0% vs. 10.1% vs. 3.7%). A *post-hoc* analysis revealed that age was positively associated with SVD severity, with individuals in the more severe SVD group being significantly older than those in the mild SVD and non-SVD groups, respectively. Furthermore, individuals in the mild SVD group were also found to be significantly older than those in the non-SVD group.

**Table 2:** Comparison between each SVD category

Variables	Normal		Mild SVD		More-severe SVD		*P* value	Arteriosclerosis/lipohyalinosis						CAA		*P* value	
	Simple SVD score 0 *n* = 324		Simple SVD score 1 *n* = 276		Simple SVD score 2 or 3 *n* = 108			SVD type 1 *n* = 246		SVD type 2 *n* = 47		SVD type 3 *n* = 56		SVD type 4 *n* = 35			
Age, years	57.80 ± 5.87		64.71 ± 8.36		68.94 ± 8.59		< 0.001	65.63 ± 8.12		67.86 ± 9.01		66.41 ± 9.98		64.35 ± 9.01		< 0.001	
Gender							0.060									0.206	
Male	129 (39.8%)		135 (48.9%)		52 (48.1%)			119 (48.4%)		22 (46.8%)		29 (51.8%)		17 (48.6%)			
Female	195 (60.2%)		141 (51.1%)		56 (51.9%)			127 (51.6%)		25 (53.2%)		27 (48.2%)		18 (51.4%)			
HTN	75 (23.1%)		109 (39.5%)		61 (56.5%)		< 0.001	106 (43.1%)		23 (48.9%)		30 (53.6%)		11 (31.4%)		< 0.001	
DM	23 (7.1%)		52 (18.8%)		25 (23.1%)		< 0.001	52 (21.1%)		12 (25.5%)		9 (16.1%)		4 (11.4%)		< 0.001	
Dyslipidemia	42 (13.0%)		28 (10.1%)		4 (3.7%)		0.024	23 (9.3%)		4 (8.5%)		3 (5.4%)		2 (5.7%)		0.279	
Cigarette smoking	44 (13.6%)		44 (15.9%)		18 (16.7%)		0.625	39 (15.9%)		10 (21.3%)		7 (12.5%)		6 (17.1%)		0.643	
Homocysteine, umol/L	11.24 (9.40-13.38)		12.50 (10.80–15.40)		13.00 (10.63–15.85)		< 0.001	12.70 (10.80–16.10)		12.90 (10.10–15.90)		12.30 (10.63–15.08)		12.40 (10.20–14.80)		< 0.001	
hsCRP, mg/dL	0.051 (0.020–0.142)		0.077 (0.030–0.169)		0.072 (0.026–0.190)		0.005	0.077 (0.031–0.172)		0.074 (0.039–0.205)		0.094 (0.022–0.173)		0.053 (0.022–0.138)		0.015	

Continuous variable: Kruskal Wallis test; Category variable: Pearson’s chi-squared test; N (%) or mean ± standard deviation or median (interquartile range)

CAA: cerebral amyloid angiopathy; DM: diabetes mellitus; HTN: hypertension; hsCRP: high-sensitivity C-reactive protein; SVD: cerebral small vessel disease

Statistically significant differences in age and the prevalence of hypertension and DM across different SVD subtype groups were also observed (Table 2, **right**). Further *post-hoc* analyses revealed that individuals with arteriosclerosis/lipohyalinosis and CAA-related SVD were older than those without SVD. Additionally, the arteriosclerosis/lipohyalinosis subtype had the highest proportions of hypertension and DM among these groups.

Importantly, no differences were found in the distributions of SVD severities and subtypes between men and women (Table 2). A marginal sex-specific discrepancy was observed in the distribution of SVD-severity groups, with a slightly higher proportion of women than men being categorized as non-SVD (60.2% vs. 39.8%, *p* = 0.06) based on SVD scores.

### Associations between circulatory inflammatory markers and covert SVD

The intra- and inter-assay coefficients of variation for homocysteine were 2.3–4.4% and 1.5–5.2%, respectively, while for hsCRP, they were 0.4–1.2% and 0.4–0.9%, respectively.

The results of the comparison between SVD-severity categories indicated that there were statistically significant differences in the levels of both circulatory markers of vascular (homocysteine) and systemic inflammation (hsCRP) across groups (Table 2, **left**). Further *post-hoc* analysis revealed that individuals with mild and more severe SVD had higher levels of circulatory homocysteine when compared to those without SVD.

The comparison of SVD subtypes also yielded statistically significant differences in the levels of homocysteine and hsCRP across groups (Table 2, **right**). In particular, *post-hoc* analyses demonstrated that individuals with arteriosclerosis/lipohyalinosis SVD had significantly higher levels of both systemic (SVD type 1 versus non-SVD) and vascular (SVD types 1 and 2 versus non-SVD) inflammatory markers than the non-SVD group, whereas CAA SVD did not differ significantly from non-SVD group.

### Sex differences in the associations between Risk factors and risks of SVD

**Fig. 3:**
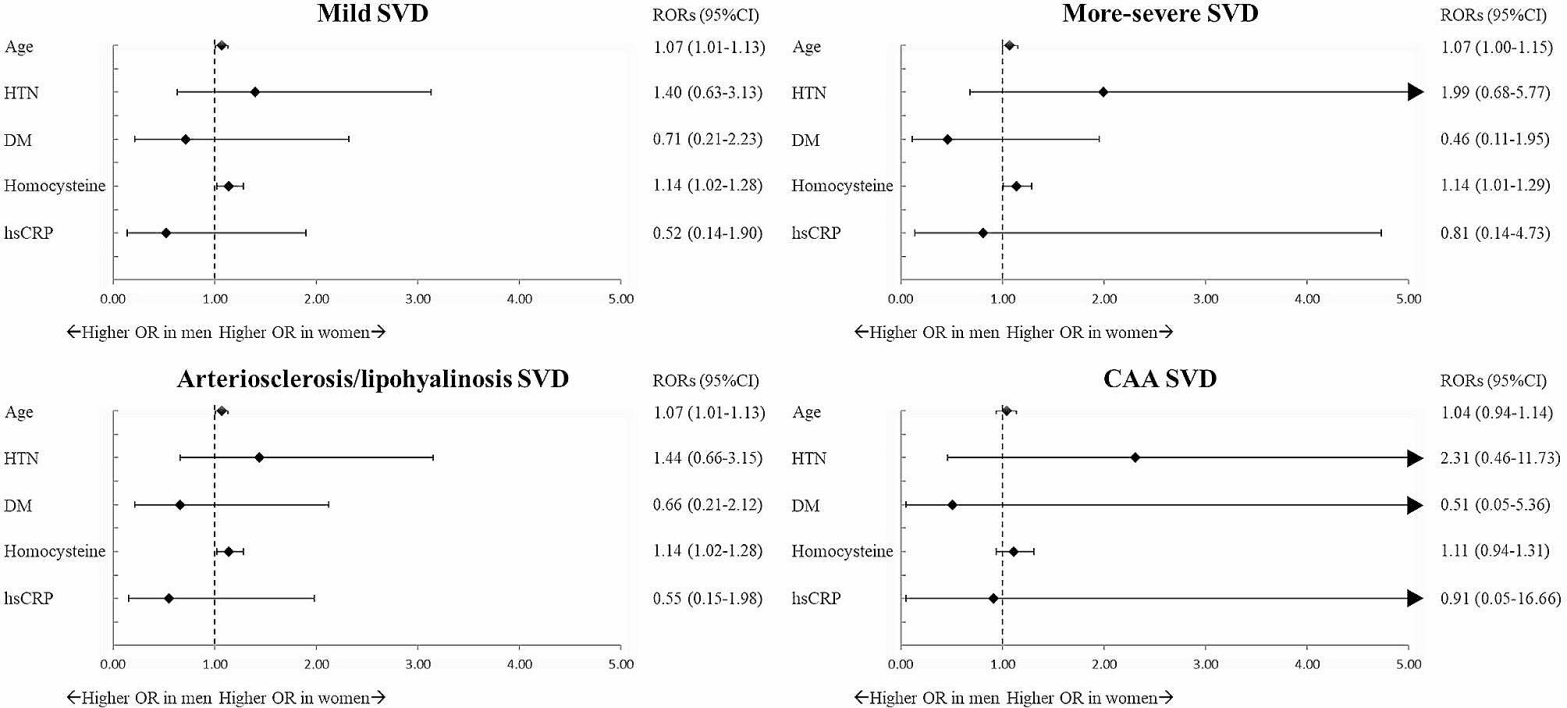
Women-to-men ratios of odds ratios for covert cerebral small vessel disease (SVD)-associated risk factors Horizontal lines indicate the corresponding 95% confidence intervals (CIs) around the ratio of odds ratios (RORs).

To examine the association between risk factors and covert SVD while controlling for potential confounding variables, we conducted a sex-specific multivariate logistic regression analysis. The findings revealed that advanced age was a significant risk factor for higher SVD severity and for all etiology in both men and women (Table 3). Furthermore, the adjusted ORs of SVD in women compared to men, after controlling for other variables, demonstrated that age imparted a higher risk of mild SVD and arteriosclerosis/lipohyalinosis SVD among women (Fig. 3).

**Table 3:** Sex-specific multiple logistic regression analysis

	SVD severity (versus non-SVD)						SVD etiology (versus non-SVD)					
	Mild			More severe			Arteriosclerosis/lipohyalinosis			Cerebral amyloid angiopathy		
Men												
Variables	Odds ratio	95% confidence interval	P value	Odds ratio	95% confidence interval	P value	Odds ratio	95% confidence interval	P value	Odds ratio	95% confidence interval	P value
Age	1.09	1.05–1.13	< 0.001	1.15	1.10–1.20	< 0.001	1.10	1.07–1.14	< 0.001	1.10	1.03–1.17	0.004
HTN	1.26	0.71–2.23	0.435	1.97	0.94–4.15	0.073	1.49	0.86–2.59	0.156	0.71	0.21–2.41	0.582
DM	2.29	0.94–5.59	0.068	2.84	0.99–8.19	0.053	2.50	1.05–5.96	0.039	1.63	0.30–8.72	0.568
Dyslipidemia	0.38	0.11–1.24	0.108	0.59	0.11–3.26	0.547	0.47	0.16–1.38	0.168	-	-	-
Homocysteine	1.01	0.96–1.07	0.662	1.00	0.93–1.08	0.956	1.01	0.95–1.07	0.745	1.02	0.92–1.12	0.752
hsCRP	1.52	0.64–3.60	0.342	0.82	0.23–2.94	0.758	1.43	0.61–3.38	0.415	0.70	0.08–6.17	0.751
Women												
Variables	Odds ratio	95% confidence interval	P value	Odds ratio	95% confidence interval	P value	Odds ratio	95% confidence interval	P value	Odds ratio	95% confidence interval	P value
Age	1.16	1.11–1.21	< 0.001	1.23	1.17–1.30	< 0.001	1.18	1.13–1.23	< 0.001	1.14	1.06–1.23	< 0.001
HTN	1.76	1.00-3.09	0.048	3.92	1.82–8.43	< 0.001	2.15	1.24–3.73	0.006	1.64	0.56–4.77	0.364
DM	1.62	0.74–3.55	0.230	1.29	0.48–3.51	0.613	1.65	0.76–3.59	0.209	0.83	0.16–4.33	0.826
Dyslipidemia	1.88	0.94–3.76	0.075	0.37	0.08–1.82	0.223	1.65	0.81–3.36	0.166	1.11	0.24–5.28	0.900
Homocysteine	1.15	1.05–1.27	0.004	1.14	1.03–1.27	0.012	1.15	1.05–1.27	0.004	1.13	0.99–1.29	0.078
hsCRP	0.79	0.30–2.08	0.631	0.66	0.19–2.23	0.503	0.78	0.30–2.04	0.617	0.64	0.09–4.44	0.651

DM: diabetes mellitus; HTN: hypertension; hsCRP: high-sensitivity C-reactive protein; SVD: cerebral small vessel disease

Conversely, the study found that only women, but not men, exhibited significant associations between hypertension and SVD (Table 3). Women with hypertension showed higher risks of developing more severe SVD and arteriosclerosis/lipohyalinosis SVD, but not CAA SVD (Table 3). Although there was a trend indicating a stronger association between hypertension and the risk of more severe SVD and arteriosclerosis/lipohyalinosis SVD in women than in men, the multivariate adjusted women-to-men RORs linked to hypertension were 1.40 (0.63–3.13) for mild SVD, 1.99 (0.68–5.77) for more severe SVD, and 1.44 (0.66–3.15) for arteriosclerosis/lipohyalinosis SVD (Fig. 3).

A noteworthy trend was observed, whereby DM seemed to be more strongly linked with a heightened risk of more severe SVD and arteriosclerosis/lipohyalinosis SVD in men than women. In particular, men with DM exhibited higher risks of more severe SVD, as well as of arteriosclerosis/lipohyalinosis SVD (Table 3). Meanwhile, the multivariate adjusted women-to-men RORs associated with DM were 0.71 (0.21–2.23) for mild SVD, 0.46 (0.11–1.95) for more severe SVD and 0.66 (0.21–2.12) for arteriosclerosis/lipohyalinosis SVD, indicating a less pronounced association between DM and SVD in women than men (Fig. 3). Furthermore, sex-specific multivariate logistic regression analysis revealed that there was no significant association between dyslipidemia and SVD in both men and women (Table 3).

### Sex differences in the associations between circulatory inflammatory markers and covert SVD

In the multivariate logistic regression models, significant associations between vascular inflammatory markers and SVD were observed exclusively in women, and not in men (Table 3). Women with elevated levels of circulatory homocysteine displayed a higher risk of SVD, as well as of arteriosclerosis/lipohyalinosis SVD **(**Table 3**)**. Moreover, the association of vascular inflammatory marker with SVD, particularly with arteriosclerosis/lipohyalinosis SVD, was found to be stronger in women than in men, with multivariate-adjusted women-to-men RORs for mild SVD, more severe SVD, and arteriosclerosis/lipohyalinosis SVD being 1.14 (1.02–1.28), 1.14 (1.01–1.29), and 1.14 (1.02–1.28), respectively (Fig. 3). The findings from sex-specific multiple logistic regression analysis adjusting for age and other vascular risk factors revealed that systemic inflammatory marker was not independently associated with SVD in either sex, as demonstrated in Table 3.

## Discussion

The relationship between inflammation and SVD with regards to sex difference has not been studied yet. Our findings revealed that, in individuals aged 50 years and above who were free of stroke and dementia, there was a sex-specific relationship between vascular inflammation and SVD. Specifically, women but not men had a higher risk of more severe and arteriosclerosis/lipohyalinosis asymptomatic SVD with increased levels of circulatory homocysteine. Conversely, our study did not identify any significant association between systemic inflammation and preclinical SVD.

Several studies have explored the potential link between circulatory inflammatory markers and SVD, yet the findings have been inconsistent [3, 15]. A recent systematic review comprising 82 articles focused on stroke or healthy populations. The majority of these studies evaluated only one SVD marker, predominantly WMH or lacune, and utilized either systemic or inflammatory markers. Notably, consistent with our study, the systemic review suggests that markers of vascular inflammation are more strongly associated with WMH or lacunes than systemic inflammation, especially in stroke patients [3]. As to the non-stroke dementia-free population, of all the markers of vascular inflammation, homocysteine has been the most extensively studied and has demonstrated the most consistent positive associations with SVD [8, 15–21]. However, homocysteine was found to be uncorrelated with SVD markers in approximately one-third of these studies [15–21]. We argue that our sex-specific findings, along with variations in study population characteristics, diagnostic tools, and SVD criteria, may contribute to conflicting results in previous studies. The current study revealed that in the preclinical stage of SVD, only women demonstrated a significant correlation between the circulatory level of homocysteine and SVD. Since men have a higher level of plasma homocysteine than women [17–19, 21], which was also shown in our study, the susceptibility to SVD with higher level of homocysteine in women might confound the associations between homocysteine and preclinical SVD in studies pooling both men and women’s data. The other possibility is that it might need a longer duration of homocysteine effects in men to show positive associations with SVD, i.e., at a later stage of disease when stroke events occur. We still require additional longitudinal research considering the sex difference to validate these postulations. Additionally, we acknowledge another possible explanation for the current sex-specific findings. It is conceivable that the absolute increase in homocysteine concentration may hold greater significance in women, considering it represents a higher increase relative to the normal range than observed in men.

In our study, after adjusting for age and other cardiovascular risk factors, we did not observe any significant associations between systemic inflammatory markers and covert SVD in both men and women. Similarly, most prior cross-sectional studies also reported negative findings in the relationship between circulatory CRP levels and SVD [3, 15, 22]. One study that explored the association between common variations in CRP and the presence of WMH and lacune in older individuals also reported negative results [23]. Despite the lack of significant association between systemic inflammatory marker and covert SVD in this current study, some longitudinal studies have demonstrated that elevated baseline circulatory CRP levels can predict subsequent SVD severity and progression [24, 25].Taking into consideration our results and those of previous studies, it appears that if systemic inflammation does play a role in the pathogenesis of SVD, its impact is likely to be less pronounced than that of vascular inflammation during the initial stages of the disease, and more substantial as the disease progresses. In addition, an alternative interpretation could be that CRP may be too simplistic as an indicator of systemic inflammation. Additional markers are necessary to corroborate the present negative findings concerning the association between systemic inflammation and covert SVD.

Several studies also performed region-specific analyses of CMBs to investigate the relationship between inflammation and the etiology of SVD, and most found that the correlation with circulatory inflammatory markers was stronger in deep or infratentorial CMBs compared to strictly lobar CMBs [1, 3, 11, 16, 19, 22, 26]. These findings are consistent with our study, suggesting that inflammation is involved in the mechanism of arteriosclerosis/lipohyalinosis SVD but not CAA, at least at the preclinical stage.

Homocysteine has been shown to act as both a marker and an element involved in the mechanism of vascular inflammation, with numerous preclinical studies demonstrating its proinflammatory effects on the vascular endothelium [27–30]. Homocysteine can also cause endothelial dysfunction through other mechanisms such as collagen accumulation, oxidative stress, decreased nitric oxide production, and epigenetic alterations [31]. Additionally, high homocysteine levels can lead to abnormal secretion of matrix metalloproteinases and transmembrane proteins, resulting in disrupted endothelial junctions and blood-brain barrier leakage [32, 33]. These findings might be the biology mechanisms underlying the association between elevated circulatory levels of homocysteine and covert SVD in women. However, whether sex differences exist in the homocysteine-related endothelial pathophysiological processes remains unexplored. As our findings indicate that homocysteine susceptibility to SVD differs between men and women, further research is needed to uncover the potential sex-specific mechanisms and management strategies for homocysteine-related vascular diseases.

Age and hypertension are the leading risk factors for SVD, particularly arteriosclerosis/lipohyalinosis etiology [1]. In our study, these factors had a stronger association with preclinical SVD in women than men. While both sexes had a higher risk of preclinical SVD with age, the risk was greater in women. These findings are consistent with previous literature that showed older women having more WMH than older men [34–36]. A recent large-scale study, the Rhineland Study, investigated the relationship between sex and WMH load across a wide age range (30–95 years) [37]. Their findings supported our results, showing that the impact of age on WMH burden is more pronounced in women than men. Moreover, they discovered that menopause may be a contributing factor to this stronger age effect in women. Regrettably, we did not collect information on menopause status in our cohort, and hence, could not explore this potential sex hormone-related mechanism in our study.

Sex differences in hypertension and its association with cardiovascular diseases have been a topic of interest in the medical community. A number of studies have found that hypertension may have a stronger association with cardiovascular disease including stroke in women [38, 39]. In particular, our study found significant associations between hypertension and preclinical SVD only in women, adding to the literature on potential sex differences in the hazard effects of vascular risk factors to cerebrovascular disease. Our finding of a greater risk of hypertension-associated SVD in women than men is in line with the results of the Rhineland Study [37], which demonstrated a higher WMH burden in women with uncontrolled hypertension compared to men, and showed that the difference could not be attributed to menopause status. The etiology behind the heightened vulnerability of women with hypertension to SVD appears to extend beyond hormonal factors [37]. Given that hypertension is often accompanied by elevated levels of homocysteine [40, 41], it is worth considering whether the sex differences in the association between homocysteine and SVD revealed in our study may explain, at least in part, the higher susceptibility of women to hypertension-related SVD. In other words, the pathophysiology associated with homocysteine may underlie and elucidate the sex-specific impact of hypertension on SVD.

This study’s strengths lie in its sample size, high-resolution brain MRI imaging, and integration of three common MRI markers to provide a comprehensive evaluation of SVD. As a non-stroke dementia-free community-based population was studied with a much less severity of SVD, the findings shed light on the very early mechanism of SVD. However, the mild SVD lesions in the population may limit the ability to show a dose-dependent association with homocysteine. While our study has made significant strides in the understanding of the relationship between inflammation and SVD, there are some limitations that should be acknowledged. First, our stratification approach using CMB locations to differentiate SVD phenotypes may be challenging in populations with coexisting CAA and arteriosclerosis/lipohyalinosis, or more advanced SVD stages where CMB distribution may extend beyond lobar subcortical areas. Second, we did not assess the severity of dilated PVS due to the lack of T2-weighted MRI imaging. Moreover, the menopause status of our female population was not taken into account, which precludes us from clarifying whether menopause is an underlying factor behind our sex-specific findings. Third, as our study participants were non-stroke dementia-free community-dwelling older adults, the number of participants in the most severe SVD category is very small (*n* = 25). We thus have grouped those with simple SVD score 2 and 3 together, future researches that can including more study participants in the most severe SVD category may provide more clinical insights. Last but not the least, despite adjusting our analyses for several confounding factors, residual confounding cannot be completely ruled out in any observational cross-sectional study.

## Conclusion

In summary, our study suggests that elevated levels of homocysteine, a marker of vascular inflammation, is associated with arteriosclerosis/lipohyalinosis SVD in women but not men at an early, preclinical stage of SVD. The underlying mechanisms for this sex-specific effect warrant further investigation and may inform more precise and personalized management of SVD.

### Electronic supplementary material

Below is the link to the electronic supplementary material.

Supplementary Material 1

## Data Availability

No datasets were generated or analysed during the current study.

## Abbreviations

SVD: Small Vessel Disease
CAA: Cerebral Amyloid Angiopathy
WMH: White Matter Hyperintensity
CMB: Cerebral Microbleeds
PVS: Dilated Perivascular Space
ILAS: I-Lan Longitudinal Aging Study
hsCRP: high-sensitivity C-Reactive Protein
TIV: Total Intracranial Volume
OR: Odds Ratio
RORs: Ratio of ORs
DM: Diabetes Mellitus
