# Supplementary material for: Sex-specific implications of inflammation in covert cerebral small vessel disease
Source: BMC Neurol. 2024 Jun 27;24:220. doi: 10.1186/s12883-024-03730-z (PMC11210151; doi:10.1186/s12883-024-03730-z)
Supplement: Supplementary file 1 — Supplementary Material 1 [file 12883_2024_3730_MOESM1_ESM.docx]

**Supplementary methods**

***Vascular Risk Factors***

Vascular risk factors were ascertained through self-report or direct measurement. Hypertension was defined as a self-reported current use of antihypertensive medication or measured systolic blood pressure ≥ 140 mmHg or diastolic blood pressure ≥ 90 mmHg. Diabetes mellitus (DM) was defined as self-reported current diabetic treatment or measured hemoglobin A1c ≥ 6.5%. Dyslipidemia was noted if there was self-report of statin use or a total cholesterol level ≥ 240 mg/dL.

***Brain MRI Acquisition***

The acquisition of multimodal neuroimaging data was carried out at the National Yang Ming Chiao Tung University to obtain information about cerebrovascular disease markers, which included WMH, lacunes, and CMBs. All MRI scans were conducted using a 3 Tesla Siemens MRI scanner (Siemens Magnetom Tim Trio, Erlangen, Germany) equipped with a 12-channel phased-array head coil. The imaging protocol consisted of a T1-weighted magnetization-prepared rapid-acquisition gradient echo sequence, a two-dimensional T2-weighted fluid-attenuated inversion recovery (FLAIR) multishot turbo-spin-echo sequence, and a three-dimensional susceptibility-weighted images (SWI) sequence. The scans were performed without gaps between slices or interpolation. The T1-weighted images were acquired with the following parameters: TR/TE/TI = 3500/3.5/1100 ms, flip angle = 7, NEX = 1, FOV = 256×256 mm, matrix size = 256×256, 192 sagittal slices, and voxel size = 1.0 mm3. The T2-weighted FLAIR images were acquired with parameters of TR/TE/TI = 9000/143/2500 ms, flip angle = 130°, NEX = 1, FOV = 220×220 mm, matrix size = 320×320, echo train length = 35, 63 axial slices, and voxel size = 0.69 mm×0.69 mm×2.0 mm. The SWI sequence was acquired with parameters of TR/TE = 28/21 ms, flip angle = 15, FOV = 256×224 mm, matrix size = 256×224, 88 axial slices, bandwidth = 120 Hz/Px, and voxel size = 1.0×1.0×2.0 mm. Before pre-processing the data, a skilled neuroradiologist reviewed all MRI scans to exclude data with significant motion artifacts or major brain abnormalities, such as trauma, tumors, intracerebral hemorrhages, or territorial infarct lesions.[1]

***Volume Quantification of WMH***

The volumetric data was estimated through the use of a well-established analytical framework, which was conducted with the Statistical Parametric Mapping (SPM12, version 7487) and Matlab R2016a software. The default settings were employed throughout the process. Initially, each participant's T2-weighted FLAIR scan was registered to the corresponding T1-weighted scan. Next, the Lesion Segmentation Toolbox (LST, version 3.0.0) was utilized to generate a native T1 space probability map of WMH, encompassing gray matter, white matter, cerebrospinal fluid, and WMH, as well as lesion-filled T1 images. Following this, the lesion-filled T1 anatomical scans were processed using the Diffeomorphic Anatomical Registration Through Exponentiated Lie Algebra–voxel-based morphometry (DARTEL-VBM) approach, which produced the corresponding deformation field. Lastly, subject-specific deformation fields were used to convert the native T1 space tissue probability maps to the standard Montreal Neurological Institute space, and the information was adjusted with total intracranial volume (TIV) to obtain the absolute volumetric data.

***Detection and Assessment of Other MRI SVD Markers***

CMBs refer to small, well-circumscribed, rounded or oval-shaped, hypointense lesions in the brain parenchyma that measure less than 10 mm in size on SWI. These lesions are distinguished from microbleed mimics, including vessels, calcifications, partial volume effects, air-bone interfaces, and hemorrhages located within or adjacent to an infarct, through rigorous exclusion criteria. The microbleed anatomical rating scale, a validated tool for measuring the quantity, topographical distribution, and location of CMBs, was utilized in our analysis.[2] The reliability of intra-rater measurements was evaluated by independently assessing CMBs in 20 randomly selected images at a separate time (K, 0.83; 95% CI, 0.79–0.90). Furthermore, we reevaluated CMBs in the 25 randomly selected images previously assessed by Dr. Chung and another investigator to ensure accuracy (K, 0.82; 95% CI, 0.79–0.88). CMBs were categorized as deep, infratentorial, or lobar based on their location. Lobar CMBs were evaluated in the frontal, parietal, temporal, and occipital regions, whereas deep regions included the basal ganglia, thalamus, internal capsule, external capsule, corpus callosum, and deep/periventricular WM. The infratentorial regions comprised the brainstem and cerebellum. Participants with CMBs were further classified into two types: strictly lobar (CMBs exclusively located in lobar regions) and mixed CMB (CMBs located in deep and/or infratentorial regions with or without lobar CMBs). Lacunes were evaluated based on high-resolution T2-weighted FLAIR anatomical scans. Specifically, we defined lacunes as fluid-filled cavities that are round or ovoid in shape, with a diameter less than 15 mm, located in the subcortical deep gray or white matter regions, and associated with adjacent WMH, as assessed using T2-weighted FLAIR anatomical scans.[3] The reliability of intra-rater measurements was evaluated by independently assessing lacune in 20 randomly selected images at a separate time (K, 0.95; 95% CI, 0.90–0.98). Furthermore, we reevaluated lacune in the 25 randomly selected images previously assessed by Dr. Chung and another investigator to ensure accuracy (K, 0.88; 95% CI, 0.85–0.90).

**References**

1. Chou KH, Lee PL, Peng LN, Lee WJ, Wang PN, Chen LK, Lin CP, Chung CP: **Classification differentiates clinical and neuroanatomic features of cerebral small vessel disease**. *Brain Commun* 2021, **3**(2):fcab107.

2. Gregoire SM, Chaudhary UJ, Brown MM, Yousry TA, Kallis C, Jäger HR, Werring DJ: **The Microbleed Anatomical Rating Scale (MARS): reliability of a tool to map brain microbleeds**. *Neurology* 2009, **73**(21):1759-1766.

3. Wardlaw JM, Smith EE, Biessels GJ, Cordonnier C, Fazekas F, Frayne R, Lindley RI, O'Brien JT, Barkhof F, Benavente OR *et al*: **Neuroimaging standards for research into small vessel disease and its contribution to ageing and neurodegeneration**. *Lancet Neurol* 2013, **12**(8):822-838.
